# Should RECOVERY have used response adaptive randomisation? Evidence from a simulation study

**DOI:** 10.1186/s12874-022-01691-w

**Published:** 2022-08-06

**Authors:** Tamir Sirkis, Benjamin Jones, Jack Bowden

**Affiliations:** 1 University of Exeter College of Medicine and Health, Exeter, UK; 2 NIHR ARC South West Peninsula (PenARC), College of Medicine and Health, University of Exeter, Exeter, England

**Keywords:** COVID-19, RECOVERY, Response-adaptive randomization, REMAP-CAP, Coronavirus, Adaptive trial, Platform trial, Simulation

## Abstract

**Background:**

The Randomised Evaluation of COVID-19 Therapy (RECOVERY) trial is aimed at addressing the urgent need to find effective treatments for patients hospitalised with suspected or confirmed COVID-19. The trial has had many successes, including discovering that dexamethasone is effective at reducing COVID-19 mortality, the first treatment to reach this milestone in a randomised controlled trial. Despite this, it continues to use standard or ‘fixed’ randomisation to allocate patients to treatments. We assessed the impact of implementing response adaptive randomisation within RECOVERY using an array of performance measures, to learn if it could be beneficial going forward. This design feature has recently been implemented within the REMAP-CAP platform trial.

**Methods:**

Trial data was simulated to closely match the data for patients allocated to standard care, dexamethasone, hydroxychloroquine, or lopinavir-ritonavir in the RECOVERY trial from March-June 2020, representing four out of five arms tested throughout this period. Trials were simulated in both a two-arm trial setting using standard care and dexamethasone, and a four-arm trial setting utilising all above treatments. Two forms of fixed randomisation and two forms of response-adaptive randomisation were tested. In the two-arm setting, response-adaptive randomisation was implemented across both trial arms, whereas in the four-arm setting it was implemented in the three non-standard care arms only. In the two-arm trial, randomisation strategies were performed at the whole trial level as well as within three pre-specified patient subgroups defined by patients’ respiratory support level.

**Results:**

All response-adaptive randomisation strategies led to more patients being given dexamethasone and a lower mortality rate in the trial. Subgroup specific response-adaptive randomisation reduced mortality rates even further. In the two-arm trial, response-adaptive randomisation reduced statistical power compared to FR, with subgroup level adaptive randomisation exhibiting the largest power reduction. In the four-arm trial, response-adaptive randomisation increased statistical power in the dexamethasone arm but reduced statistical power in the lopinavir arm. Response-adaptive randomisation did not induce any meaningful bias in treatment effect estimates nor did it cause any inflation in the type 1 error rate.

**Conclusions:**

Using response-adaptive randomisation within RECOVERY could have increased the number of patients receiving the optimal COVID-19 treatment during the trial, while reducing the number of patients needed to attain the same study power as the original study. This would likely have reduced patient deaths during the trial and lead to dexamethasone being declared effective sooner. Deciding how to balance the needs of patients within a trial and future patients who have yet to fall ill is an important ethical question for the trials community to address. Response-adaptive randomisation deserves to be considered as a design feature in future trials of COVID-19 and other diseases.

**Supplementary Information:**

The online version contains supplementary material available at 10.1186/s12874-022-01691-w.

## Background

Coronavirus disease 2019 (COVID-19) is a condition caused by the severe acute respiratory syndrome coronavirus 2 [1]. On March 11, 2020, the global incidence and virulence of COVID-19 met the criteria for the World Health Organisation to declare it a pandemic [2]. At the time of writing (April 2022), the disease has caused 500 million cases and 6 million deaths worldwide [3]. Furthermore, some patients who contracted COVID-19 report experiencing long covid, a condition consisting of many symptoms such as profound fatigue that can persist long after the initial infection has passed [4]. It has been estimated there are around 2 million people in the UK who have suffered from long covid [5]. The effects of the pandemic have been far reaching and extend beyond just those infected. For example, many patients with suspected cancer are not receiving appropriate early management, which experts believe will lead to increased mortality as their condition remains untreated [6]. Likewise, the pandemic has been noted to increase and exacerbate mental health problems such as stress and anxiety [7]. In terms of the wider societal impact, the pandemic has also led to a sharp increase in extreme poverty, 1.5 billion students to miss out on education, and increasing food insecurity [8].

When COVID-19 first emerged, very little was known about its pathophysiology and, as a result, clinicians were unsure which treatments would reduce COVID-19’s associated morbidity and mortality. To address this, large scale randomised clinical trials were quickly designed, authorised, and initiated in patients with severe COVID-19 symptoms in a bid to find effective treatments. One of the most high-profile examples is the **R**andomised **E**valuation of **COV**id-19 Th**ER**ap**Y** (RECOVERY) trial [9]. It commenced on March 19, 2020, 8 days after the pandemic announcement, with the aim of discovering new treatments that are effective in reducing 28-day mortality in patients hospitalised with confirmed or suspected COVID-19 [9]. To date, 15 treatments have been trialled, most consisting of repurposed drugs [9]. Results have been published for nine trial treatments. Most of these nine treatments, including the antimalarial drug hydroxychloroquine [10] and the antiviral combination of lopinavir-ritonavir (lopinavir) [11], were not found to have a significant effect in reducing COVID-19 mortality. However, three treatments have been declared successful: dexamethasone [12], tocilizumab [13] and REGENERON [14]. When analysing the dexamethasone data, researchers found a statistically significant effect, with fewer patients dying on dexamethasone (22.9%) compared to standard care (25.7%) [12]. A stratified analysis was also performed in three pre-specified patient subgroups who received distinct levels of respiratory support at the time of randomisation: (i) no oxygen, (ii) oxygen only, and (iii) oxygen through invasive mechanical ventilation. The subgroup analysis uncovered important treatment effect heterogeneity: Patients in both subgroups (ii) and (iii) received benefit from taking dexamethasone compared to standard care alone, with the group requiring the most respiratory support (iii) receiving the largest benefit. However, patients in group (i) who required the lowest level of respiratory support appeared to fare better with standard care (although this was not statistically significant) [12]. These results are shown in Table 1. The National Institute for Health and Care Excellence issued guidelines based on this research, stating dexamethasone should be given to hospitalised patients with COVID-19 if they require oxygen [15].

**Table 1:** Mortality data for the RECOVERY trial stratified by patient group

Patient subgroup	Number allocated standard care	Number allocated dexamethasone	Number allocated hydroxy-chloroquine	Number allocated lopinavir	Mortality on standard care	Mortality on dexamethasone	Mortality on hydroxy-chloroquine	Mortality on lopinavir
**No oxygen**	1034	501	362	425	14.0%	17.8%	16.0%	16.7%
**Oxygen-only**	2604	1279	938	1131	26.2%	23.3%	27.0%	24.7%
**Invasive ventilation**	683	324	261	60	41.4%	29.3%	42.1%	40.0%

RECOVERY is an excellent example of a modern adaptive platform trial [16]. Unlike a traditional trial where all design aspects (including the treatments to be compared and the sample size) must be decided before the trial commences, adaptive platform trials have the freedom to continue indefinitely. Whilst the trial is ongoing, new experimental treatments can be added and tested, old experimental treatments showing little benefit can be dropped and effective experimental treatments can be ‘graduated’ to become the de-facto standard of care. Many adaptive trial designs exist, but their common aim is to be more flexible and resource efficient. Supporters of adaptive designs assert that they are more ethical [17], although this is not universally accepted [18]. One of the most controversial features that can be incorporated into an adaptive trial is response-adaptive randomisation (RAR). RECOVERY did not incorporate this, preferring instead to use standard ‘fixed’ randomisation (FR) probabilities when allocating patients to experimental or standard care arms during the trial. Under a RAR scheme, allocation typically starts in a FR state, randomising patients to all trial arms with equal probability. RAR subsequently facilitates the adaptation of the allocation ratio, as interim analyses begin to show that there is a genuine difference between outcomes in the different arms, to favour treatments that have a higher estimated probability of a favourable outcome. A recent example of a COVID-19 study that uses RAR is the **R**andomised, **E**mbedded, **M**ulti-factorial, **A**daptive **P**latform Trial for **C**ommunity-**A**cquired **P**neumonia (REMAP-CAP) [19]. This is a trial that aims to evaluate multiple interventions simultaneously for community acquired pneumonia but has a sub-platform, REMAP-COVID, created to assess COVID-19 treatments [20].

The aim of this paper is to investigate, by simulation, the possible benefit of applying RAR instead of FR to assign patients to different treatment arms in the RECOVERY trial. We hypothesised that applying RAR would reduce the number of deaths amongst trial participants by allocating more patients to their optimal treatment. To implement RAR, we use the REMAP-CAP algorithm as well as our own bespoke tuning algorithm. In different simulations we apply each RAR method across the whole patient cohort, and then separately within patient subgroups (i)-(iii). We have also compared simulations covering trials with either two or four treatment arms. We apply FR using allocation ratios of 1:1 and 2:1 (with respect to standard care: dexamethasone), with the latter having been used in RECOVERY. To quantify benefit, we focus on the following five metrics:

- The proportion of patients allocated to each treatment;
- The expected number of deaths throughout trial;
- The statistical power to detect a treatment effect in all patients and in patient subgroups;
- The bias and mean squared error of the treatment effect estimate;
- The familywise type 1 error of wrongfully declaring one or more treatments as having a significant benefit

## Methods

### Simulation set up

Two simulations were set up using R statistical language [21] with parameters selected to closely resemble the observed results of the RECOVERY trial. The first of these simulations was based on two arms of the RECOVERY trial: namely the dexamethasone arm and the control arm. The original trial collected primary outcome data from a total of 6425 patients across the dexamethasone (*N* = 2104) and standard of care (*N* = 4321) arms in 81 days between March 19 and June 8, 2020. At this point the dexamethasone arm was halted and results of its efficacy were published [12]. The second simulation includes the addition of two further experimental treatments tested in RECOVERY, namely hydroxychloroquine (*N* = 1561) and lopinavir (*N* = 1616), to create a 4-arm trial simulation. Although neither of the latter treatment arms were found to have a significant benefit in reducing COVID-19 mortality, they were included in the simulations to demonstrate the very different behaviour of RAR procedures in the multi-arm (as opposed to two arm) setting.

To provide a means to implement RAR, patient outcome data was simulated for 100 days (or blocks) each consisting of 80 patients to give a total of 8000 patients in the 2-arm trial, or of 120 patients to give a total of 12,000 patients in the 4-arm trial. This sample size and trial duration intentionally exceeds that of RECOVERY, because it enables our study to evaluate the statistical properties of the design at both smaller and larger sample sizes than the actual trial. The sample size of the trial with both two-arm and four-arm simulations approximates to that of the original trial at 80 days, and most metrics are collected from this point. To match the RECOVERY study population, 24, 60 and 16% of each block were drawn from patient subgroups (i)-(iii), respectively. Patient outcomes (Y) representing the primary outcome of mortality at 28 days were generated from a Bernoulli distribution. For patient *i* randomized in block *j* in patient subgroup *k*, on treatment *l*:

$${Y}_{i,j,k,l}\sim bern\left({P}_{k,l}\right),$$

where *i* = 1, …, 80 (or 120 for the 4-arm simulation), *j* = 1, …,100, k = i, ii, iii, *l* = 0 (standard of care), 1 (standard of care + dexamethasone), 2 (standard of care + hydroxychloroquine),or 3 (standard of care + lopinavir),. The values of *P*_*k*, *l*_ (the 28 day mortality rate) match the rates observed in RECOVERY (see Table 1).

### Two arm trial: randomisation allocation strategies

Six allocation strategies (two FR and four RAR) were investigated as part of this simulation. Each strategy yielded an allocation rate, which was used in a binomial data generating function in order to create variation within the simulation and avoid rounding errors. FR was investigated using both a 1:1 and a 2:1 standard care: dexamethasone ratio. Given the underlying outcome rates assumed in each trial arm are similar (Table 1), 1:1 allocation, or a 50% probability of receiving either, drug is near-optimal in terms of statistical power according to Neyman’s rule [22] (the exact value being a 51%/49% split). For further details see Additional file 1*Technical Appendix A*. The latter 2:1 strategy was used in RECOVERY. RAR was investigated using two randomisation algorithms, our own bespoke tuning algorithm (T) and the algorithm used in REMAP-CAP (RMC). We use T_f_ and RMC_f_, to denote the RAR allocation procedures applied to trial patients across the full patient cohort. We use T_s_ and RMC_s_, to denote RAR allocation procedures applied within each patient subgroup. Specifically, the probability of patients in block *j* and subgroup k being allocated to the dexamethasone group given treatment and outcome data on all preceding patients in blocks 1, …,*j*-28 is denoted by α_j,k_, where:

$${\alpha}_{j.k}=\left\{\begin{array}{c}\frac{1}{2}:\mathrm{for}\ 1:1\ \mathrm{Fixed}\ \mathrm{equal}\ \mathrm{Randomisation}\ (FeR)\\ {}\frac{1}{3}:\mathrm{for}\ 1:2\ \mathrm{Fixed}\ \mathrm{unequal}\ \mathrm{Randomisation}\ (FuR)\\ {}\frac{\theta {(1)}^s}{\theta {(1)}^s+{\left(1-\theta (1)\right)}^s}:\mathrm{Cohort}\ \mathrm{Tuning}\ \mathrm{algorithm}\ \left({T}_f\right)\ \\ {}\sqrt{\frac{\theta (1)}{\sum_{j=1}^{J-28}{n}_{j,1}+1}}/\left(\sqrt{\frac{\theta (1)}{\sum_{j=1}^{J-28}{n}_{j,1}+1}}+\sqrt{\frac{\theta (0)}{\sum_{j=1}^{J-28}{n}_{j,0}+1}}\right):\mathrm{Cohort}\ \mathrm{REMAPCAP}\ \mathrm{algortihm}\ \left({RMC}_f\right)\\ {}\frac{\theta_k{(1)}^s}{\theta_k{(1)}^s+{\left(1-{\theta}_k(1)\right)}^s}:\mathrm{Subgroup}\ \mathrm{Tuning}\ \mathrm{algorithm}\ \left({T}_s\right)\\ {}\sqrt{\frac{\theta_k(1)}{\sum_{j=1}^{J-28}{n}_{j,k,1}+1}}/\left(\sqrt{\frac{\theta_k(1)}{\sum_{j=1}^{J-28}{n}_{j,k,1}+1}}+\sqrt{\frac{\theta_k(0)}{\sum_{j=1}^{J-28}{n}_{j,k,0}+1}}\right):\mathrm{Subgroup}\ \mathrm{REMAPCAP}\ \mathrm{algorithm}\ \left({RMC}_s\right)\end{array}\right.$$

Here, *θ*(*l*) represents the posterior probability that treatment *l* is optimal based on patients who have been in the trial for at least 28 days, either for the full cohort or for subgroup k (further details supplied in Additional file 1 Technical Appendix B); *s* is the proportion of trial stages completed at point of adaption; and *n*_*j*, *k*, *l*_represents number of patients in stage k in the cohort/sub-group that have been allocated treatment *l* .

In addition, allocation probabilities in RAR Schemes T and RMC were constrained by a maximum and minimum value according to the following rule:

$${\alpha}_{j,k}=\left\{\begin{array}{c}0.9\ \mathrm{if}\ {\alpha}_{j,k}>0.9\\ {}{\alpha}_{j,k}\ \mathrm{if}\ 0.1\le {\alpha}_{j,k}\le 0.9\ \\ {}0.1\ \mathrm{if}\ {\alpha}_{j,k}<0.1\end{array}\right.$$

Both the T and RMC RAR procedures used a “burn-in” period (a period where adaptive randomisation was not applied) for the first 34 days of trial recruitment (to allow participants recruited within the first week to reach the primary endpoint of 28 days at the point of first adaption), meaning the first 2720 patients or 42.5% of the trial were allocated in a fixed 1:1 ratio. Only after this point, new patients were allocated using RAR, with the α_j,k_ ratio being updated every 7 days. Each simulation was performed 1000 times.

### Four arm trial: randomisation allocation strategies

Four allocation strategies (two FR and two RAR) were investigated as part of this simulation.

- FR was investigated using both a 1:1:1:1 and a 2:1:1:1 standard care: dexamethasone:hydroxychloroquine:lopinavir ratio. The 2:1:1:1 ratio was the one used in the RECOVERY trial, making it the simulated strategy that is the most congruent to the original trial.
- T_f_ and RMC_f_ RAR strategies in the experimental arms, but with 40% fixed randomisation probability for the standard care group. The probability of patients in block j and subgroup k being allocated each one of the treatment groups l (2 = dexamethasone, 3 = hydroxychloroquine, 4 = lopinavir) given treatment and outcome data on all preceding patients in blocks 1, …,j-28 is denoted by α_j,k,l_, where:
  $${a}_{j,k,l}=\left\{\begin{array}{c}\frac{1}{4}:\mathrm{for}\ 1:1:1:1\ \mathrm{Fixed}\ \mathrm{equal}\ \mathrm{Randomisation}\ \left(\mathrm{FeR}\right)\\ {}\frac{1}{5}:\mathrm{for}\ 2:1:1:1\ \mathrm{Fixed}\ \mathrm{unequal}\ \mathrm{Randomisation}\ \left(\mathrm{FuR}\right)\\ {}0.6\times \frac{\theta {(l)}^s}{\theta {(l)}^s+{\left(1-\theta (l)\right)}^s}/{\sum}_{l=1}^L\frac{\theta {(l)}^s}{\theta {(l)}^s+{\left(1-\theta (l)\right)}^s}:\mathrm{Cohort}\ \mathrm{Tuning}\ \mathrm{algorithm}\ \left({\mathrm{T}}_{\mathrm{f}}\right)\\ {}0.6\times \sqrt{\frac{\theta (1)}{\sum_{j=1}^{J-28}{n}_{j,1}+1}}/{\sum}_{l=1}^L\sqrt{\frac{\theta (l)}{\sum_{j=1}^{J-28}{n}_{j,l}+1}}:\mathrm{Cohort}\ \mathrm{REMAPCAP}\ \mathrm{algortihm}\ \left({\mathrm{RMC}}_{\mathrm{f}}\right)\end{array}\right.$$

In the four-arm simulation, allocation probabilities in RAR were constrained by a maximum and minimum value according to the following rule:

$${\alpha}_{j,k,l}=\left\{\begin{array}{c}{\alpha}_{j,k,l}-\left(0.05-\min \left({\alpha}_{j,k}\right)\right)\ if\ \max \left({\alpha}_{j,k}\right)={\alpha}_{j,k,l}\ and\ \min \left({\alpha}_{j,k}\right)<0.05\\ {}{\alpha}_{j,k,l}\ if\ 0.1\le {\alpha}_{j,k,l}\le 0.9\ \\ {}0.05\ if\ {\alpha}_{j,k,l}<0.05\end{array}\right.$$

### Trial metrics

To match the sample size in the two and four arms of recovery at the point where recruitment to dexamethasone ended, single point metrics were performed at the point where 80% of the simulation was completed. The x-axes of all plots were calibrated with the sample size of recovery with 100% being *N* = 6400 patients (or *N* = 9600 for the four-arm simulation) to match the end of recruitment, and 125% being the entire length of the simulation. To assess the performance of the methods, the following summary measures were calculated across the 1000 simulated trials:

- The expected or average number of patients allocated to Dexamethasone *E*[*N*_*d*_]
- The expected or average number of deaths *E*[*N*_*Y*_] at the point where 6400 patients were allocated to a treatment, a number chosen due to its proximity to the 6425 patients recruited in RECOVERY.
- T1E: The expected probability of incorrectly rejecting the null hypothesis of no treatment effect, when all treatments have the same mortality rate, also known as the type I error rate. *E*[*t*1*err*], across the trial. In two arm settings where one null hypothesis was tested, the significance threshold of the test was fixed at 5%. In multi-arm strategies, where more than one hypothesis was tested, *p*-value ratios were adjusted using the Bonferroni correction for the specific number of hypotheses tested (i.e. the p-level threshold used was 0.05 divided by 3) in order to preserve the family wise error rate.
- Power: The expected probability of correctly rejecting the null hypothesis of no treatment effect at the end of a block. This was calculated using a logistic regression model. For the subgroup-level randomisation, power was only calculated for subgroups ii and iii, as in subgroup i dexamethasone performed worse than standard care. For the multi-arm trial simulation, this was calculated for dexamethasone and lopinavir. Although lopinavir didn’t have a significant effect when its RECOVERY results were published, both lopinavir and dexamethasone have point estimates of mortality lower than standard care group (unlike hydroxychloroquine). Therefore, to compare power across the trial for more than one treatment, it was assumed that this mortality difference was genuine and would be statistically confirmed with a larger sample size than in RECOVERY.

The relative bias and mean squared error in the treatment effect estimate. The first metric, ${bias}_k=E\left[\frac{\left({\hat{P}}_{k,0}-{\hat{P}}_{k,1}\right)-\left({P}_{k,0}-{P}_{k,1}\right)}{\left({P}_{k,0}-{P}_{k,1}\right)}\right]$, where *P*_*k*, *l*_ is the actual mortality rate and ${\hat{P}}_{k,l}$ is the estimated mortality rate in subgroup *k* (1 = (i), 2 = (ii), 3 = (iii)) on treatment *l* (1 for dexamethasone, 0 for standard of care) from the trial. This bias is only calculated for RAR procedures, as only RAR induces bias in treatment effect estimates, as explained in technical Additional file 1 appendix C. Mean squared error is calculated by $MSE=\frac{1}{N}\sum_{n=1}^N{\left(\left({\hat{P}}_{k,0}-{\hat{P}}_{k,1}\right)-\left({P}_{k,0}-{P}_{k,1}\right)\right)}^2$.

### Summary

To summarise, the two-arm simulation study investigated six treatment allocation methods:

- 1:1 FR across all patients (FeR)
- 1:2 FR across all patients (FuR)
- T algorithm across all patients (T_f_)
- RMC algorithm across all patients (RMC_f_)
- T algorithm within subgroups (i)-(iii) separately (T_s_)
- RMC algorithm within subgroups (i)-(iii) separately (RMC_s_)

In addition, the four-arm simulation study investigated the following four treatment allocation methods:

- 1:1:1:1 allocation across all patients (FeR)
- 2:1:1:1 allocation with the control group having the most allocation (FuR)
- 40% of patients allocated to the control group, with the rest allocated to a treatment arm according to the T algorithm
- 40% of patients allocated to the control group, with the rest allocated to a treatment arm according to the RMC algorithm

## Results

The operating characteristics for the two-arm and four-arm simulations are summarised below in Table 2 and Table 3 respectively.

**Table 2:** Reported operating characteristics from the two-arm simulation

Operating characteristic where sample size matches RECOVERY	FuR	FeR	T_f_	RMC_f_	T_s_			RMC_s_		
					i	ii	iii	i	ii	iii
					61.6	38.6	32.3	60.7	38.3	29.6
Proportion allocated to dexamethasone (%)	33.3	50	62.9	62.4	57.0 (average			57.8 (average)		
					38.4	61.4	67.7	39.3	61.7	70.4
Mortality rate (deaths/6400 and percentage)	1583 (24.7%)	1556 (24.3%)	1528 (23.9%)	1527 (23.9%)	1516 (23.7%)			1514 (23.7%)		
Statistical power (%)	65.2	67.7	66.7	66.1	N/A	52.3	97.4	N/A	53.9	97.6
Type 1 error rate (%)	4.7	5.3	5.0	5.0	5.8			5.2		
Bias (×10^−2^)	0	0	−2.8	−3.3	−0.7	0.8	0.7	−0.4	2.6	1.5
Mean Squared error (× 10^−4^)	1.27	1.18	1.21	1.28	6.57	3.57	16.0	6.67	3.55	16.2

**Table 3:** the operating characteristics reported from the 4-arm simulation

Operating characteristic where sample size matches RECOVERY		FuR	FeR	T_f_	RMC_f_
Proportion allocated to control (%)		40.0	25.0	40.0	40.0
Proportion allocated to dexamethasone (%)		20.0	25.0	27.0	26.6
Proportion allocated to hydroxychloroquine (%)		20.0	25.0	14.9	15.0
Proportion allocated to lopinavir (%)		20.0	25.0	18.1	18.4
Mortality (deaths/9600 and percentage)		2394 (24.9%)	2388 (24.9%)	2373 (24.7%)	2374 (24.7%)
Statistical power dexamethasone (%)		54.6	49.8	59.9	58
Statistical power lopinavir (%)		9.6	9.2	6.5	7.9
Family-wise error rate (%)		3.8	4.7	4.9	5.1
Bias (×10^−2^)	Dexamethasone	0	0	−4.1	−5.4
	Hydroxychloroquine			10.0	6.2
	Lopinavir			− 33.2	−39.1
Mean Squared error (×10^−4^)	Dexamethasone	1.60	1.98	1.50	1.57
	Hydroxychloroquine	1.70	1.94	2.28	2.11
	Lopinavir	1.84	1.89	1.81	1.91

### Allocation to each arm

**Fig. 1:**
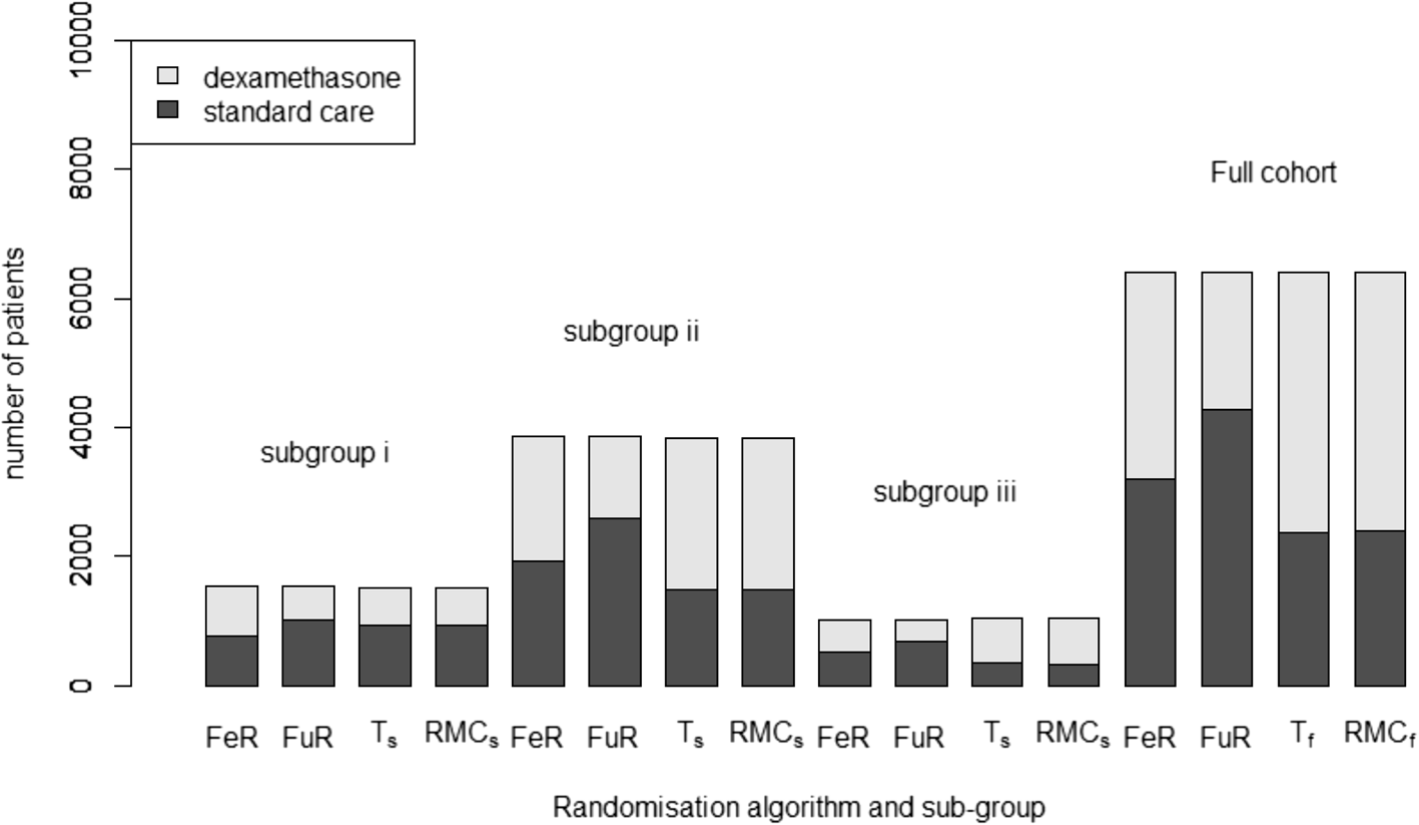
Allocation proportions within the full cohort and within each patient subgroup for all FR and RAR procedures. Horizontal blue line shows *n* = 6400, the sample size of RECOVERY

In the two-arm simulation, both cohort RAR methods led to more patients receiving dexamethasone compared to either FR approach. T_f_ led to slightly more patients receiving dexamethasone than RMC_f_. When considering subgroup-specific RAR, each subgroup has its own trend. In subgroup (i), both adaptive methods allocate slightly less patients to standard care compared to FuR, with T_s_ allocating standard care to the most patients. In subgroups (ii) and (iii), the RAR algorithms allocate mainly to dexamethasone, with RMC_s_ allocating the most patients to dexamethasone. However, the treatment allocation disparity is much larger in the subgroup (iii). These differences are demonstrated in Fig. 1.

**Fig. 2:**
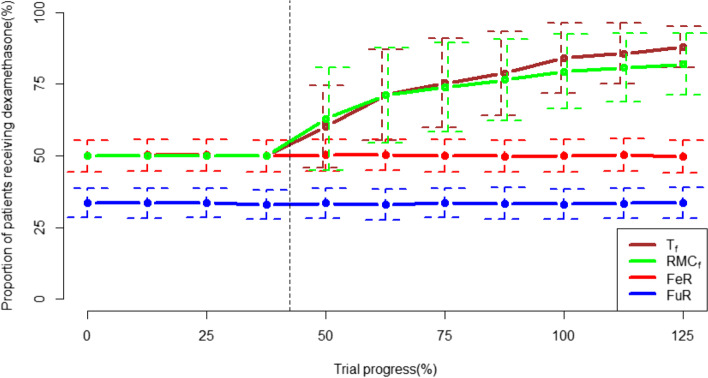
The proportion of patients receiving dexamethasone across the trial for FR and cohort-level RAR frameworks. Vertical line at 42.5% trial progress represents the end of the burn-in period, with bars dotted bars representing +/− 1 standard deviation for each allocation

**Fig. 3:**
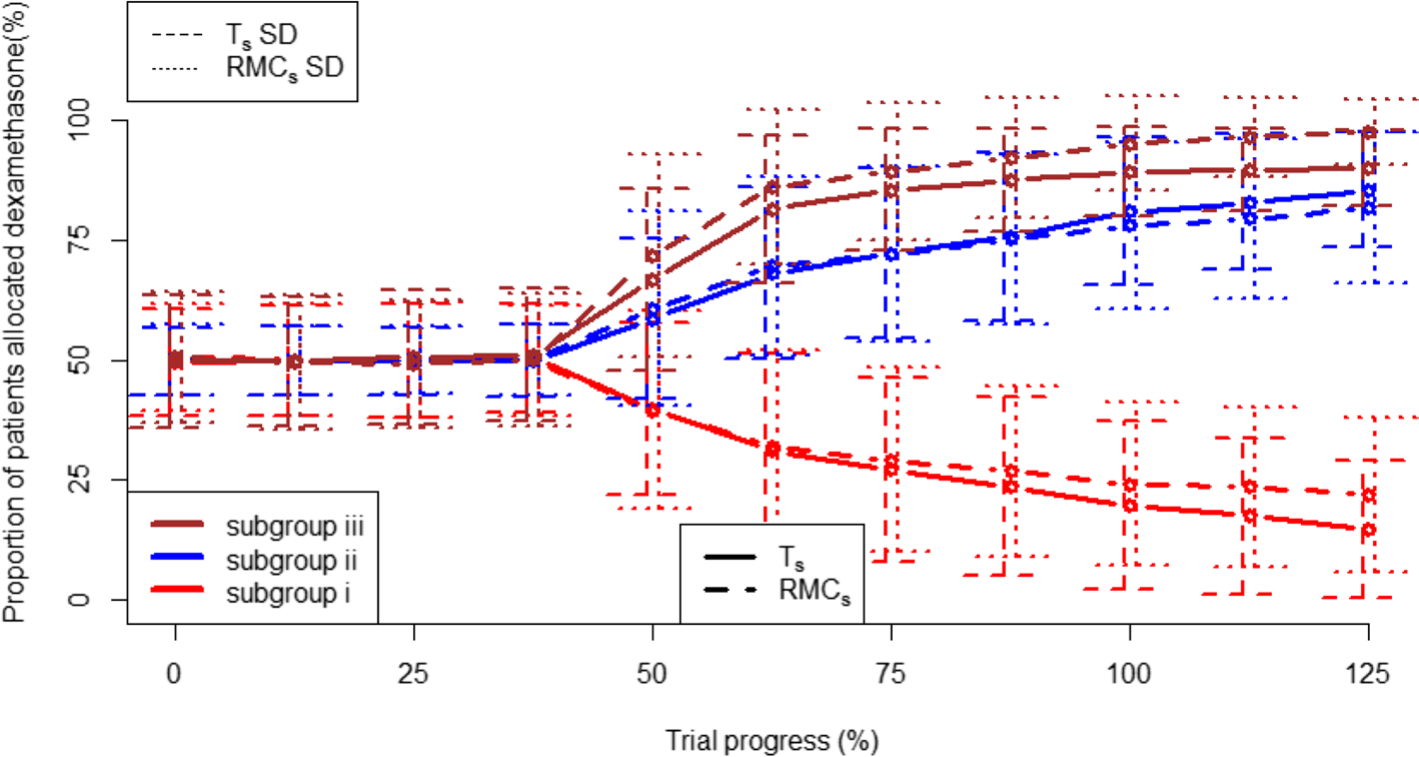
the allocation of patients to dexamethasone and no treatment in T_s_ and RMC_s_. Vertical line at 42.5% trial progress represents the end of the burn-in period, with lines +/− 1 standard deviation bars

The RAR algorithms also differ in how they “ramp up” allocation to the optimal treatment as the trial progresses. T_f_ increases randomisation to the optimal treatment at a steadier rate from the end of the burn-in period, whereas RMC_f_ increases randomisation faster in the early trial stages but also plateaus earlier. This is consistent with the pattern in subgroup-level RAR methods, T_s_ and RMC_s,_ for subgroups (i) and (ii). However, in subgroup (iii), the RMC_s_ algorithm ramps up allocation to dexamethasone faster across the whole RAR phase of the trial. This is demonstrated in Figs. 2 and 3.

**Fig. 4:**
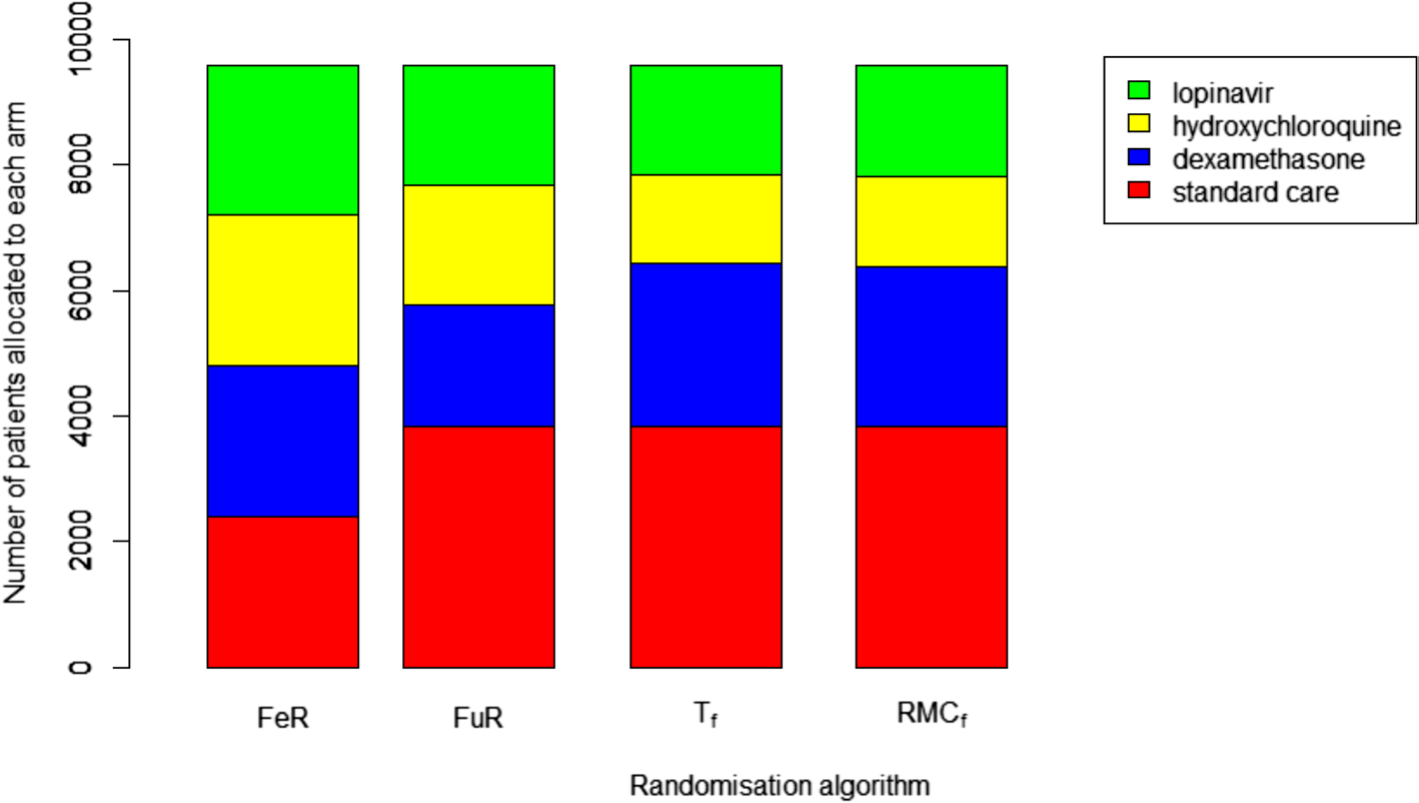
the amount of patient allocated to each trial arm under different allocation strategies

In the four-arm simulations, allocation to the control group was protected and fixed at 40%. In terms of the experimental treatment arms, RAR led to dexamethasone receiving the highest allocation of patients, followed by lopinavir, with hydroxychloroquine receiving the fewest. The T_f_ randomisation algorithm leads slightly more patients to receive dexamethasone and fewer to receive hydroxychloroquine than the RMC_f_ algorithm. These results are shown in Fig. 4.

**Fig. 5:**
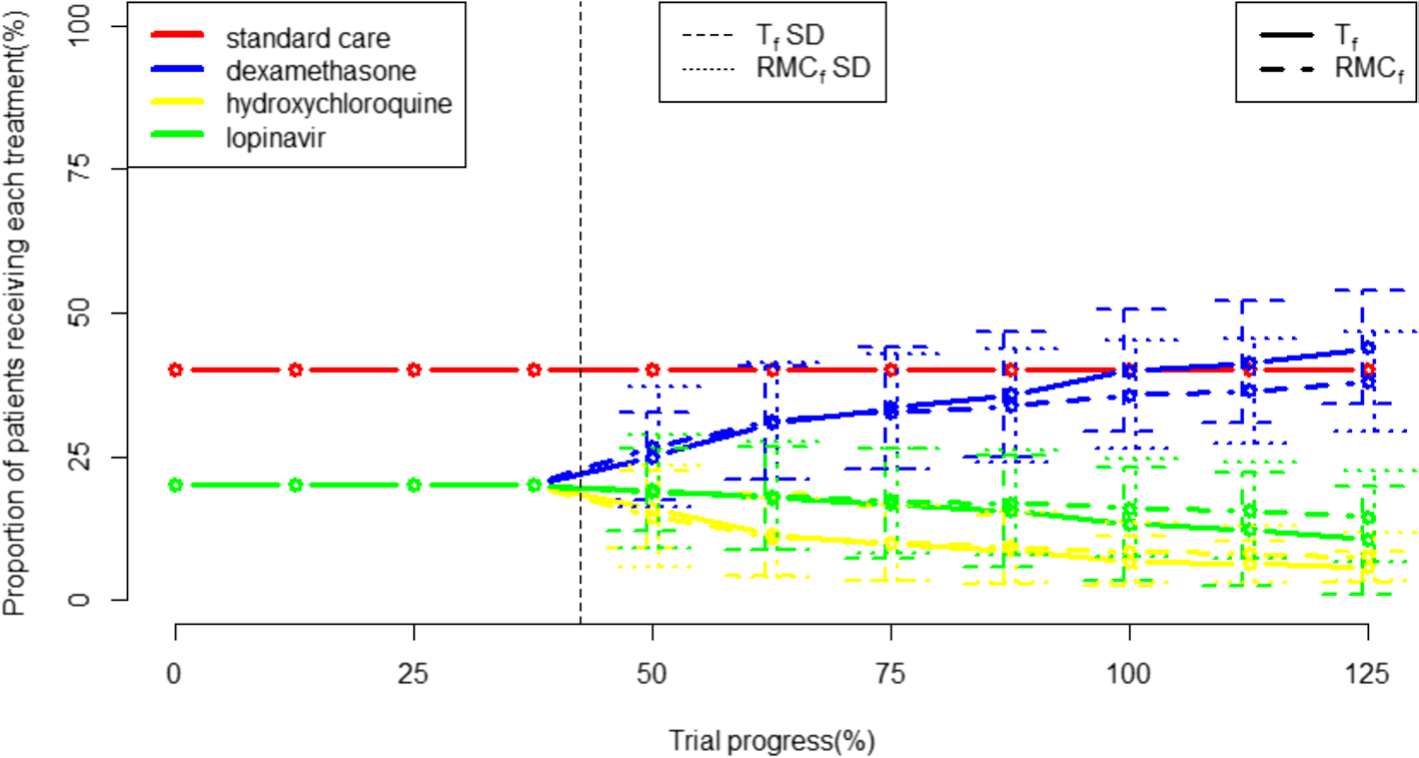
the allocation to each trial arm using each randomisation algorithm. Vertical dotted line represents the point in which adaptive randomisation begins

As soon as the burn-in period is finished, participant allocation quickly shifts to assigning more patients to dexamethasone while decreasing how many patients are assigned to hydroxychloroquine and lopinavir. Allocation to hydroxychloroquine decreases at a steeper rate than allocation to lopinavir. The divergence in allocation probabilities between dexamethasone and the other treatment arms occurs more sharply with T_f_ randomisation than with RMC_f_. The dexamethasone allocation exceeds the 40% of patients allocated to standard care at 100% trial completion using the T_f_ algorithm. This is demonstrated on Fig. 5.

### Mortality rates

In the 2-arm simulations, FR led to the highest number of expected deaths (with FuR being worse than FeR). This was followed by cohort-level RAR, with the lowest mortality rates observed when using subgroup-specific RAR. There was little difference between the randomisation algorithms, with RMC_f_ and RMC_s_ attaining a marginally lower mortality rate than T_f_ and T_s_ respectively. The expected mortality figures are given in Table 4, expressed as deaths prevented relative to FuR.

**Table 4:** Expected mortality rates in whole trial for each randomisation method, given at point that the dexamethasone arm’s results were published

Randomisation strategy	Average no. of deaths at n = 6400 +/− standard error (percentage)	Deaths prevented compared to FuR at ***n*** = 6400
FuR	1583 +/− 1.1 (24.7%)	–
FeR	1556 +/− 1.0 (24.3%)	27
T_f_	1528 +/− 1.2 (23.9%)	55
RMC_f_	1527 +/− 1.2 (23.9%)	56
T_s_	1516 +/− 1.2 (23.7%)	67
RMC_s_	1514 +/− 1.2 (23.7%)	69

In the four-arm simulations, the mortality rate decreases compared to the FuR strategy are smaller. T_f_ and RMC_f_ lead to very similar reductions in mortality, which is likewise consistent with the FeR strategy. These results are demonstrated in the Table 5.

**Table 5:** Expected mortality rates in the whole trial for each randomisation, given at the point that the results for the dexamethasone arm were published

Randomisation strategy	Average number of deaths at ***n*** = 9600 +/− standard error (percentage)	Deaths prevented compared to FuR at ***n*** = 9600
FuR	2394 +/− 1.6 (24.9%)	–
FeR	2388 +/− 1.6 (24.9%)	6
T_f_	2373 +/− 1.6 (24.7%)	21
RMCf	2374 +/− 1.7 (24.7%)	20

### Statistical power

**Fig. 6:**
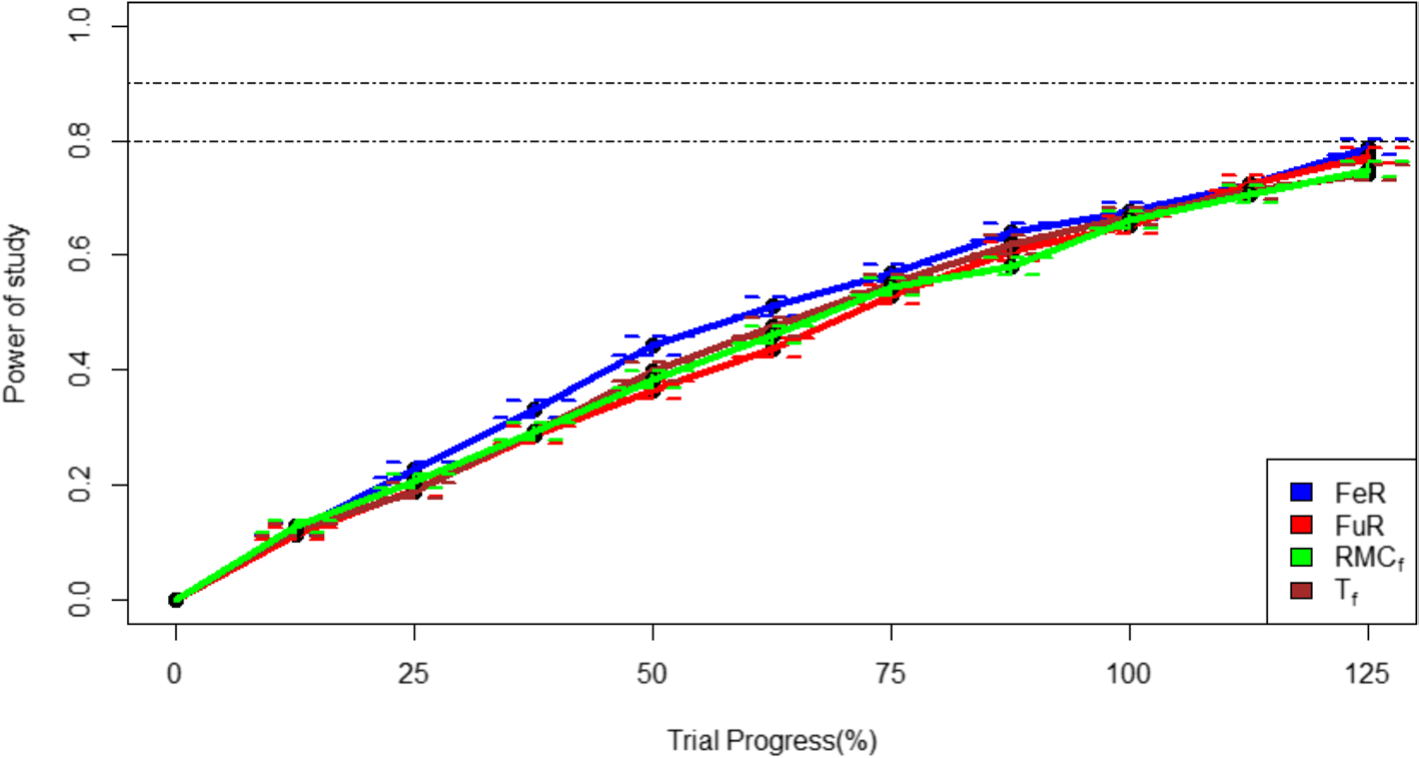
Comparing fixed randomisation and cohort-level adaptive randomisation approaches in terms of study power across the length of the trial. Bars around each point represent +/− 1 standard error in power, and horizontal lines are plotted at a power of 80 and 90%

In two-trial simulations, FeR led to the highest study power on a cohort level, as predicted by Neyman’s rule. FuR, T_f_ and RMC_f_ all performed similarly, with FuR having slightly more power by the end of the simulation and at the point where the simulation has almost the same sample size as RECOVERY. Comparing the cohort RAR algorithms, RMC_f_ performs slightly better than T_f_ in most points of the trial. These results are shown in Fig. 6.

**Fig. 7:**
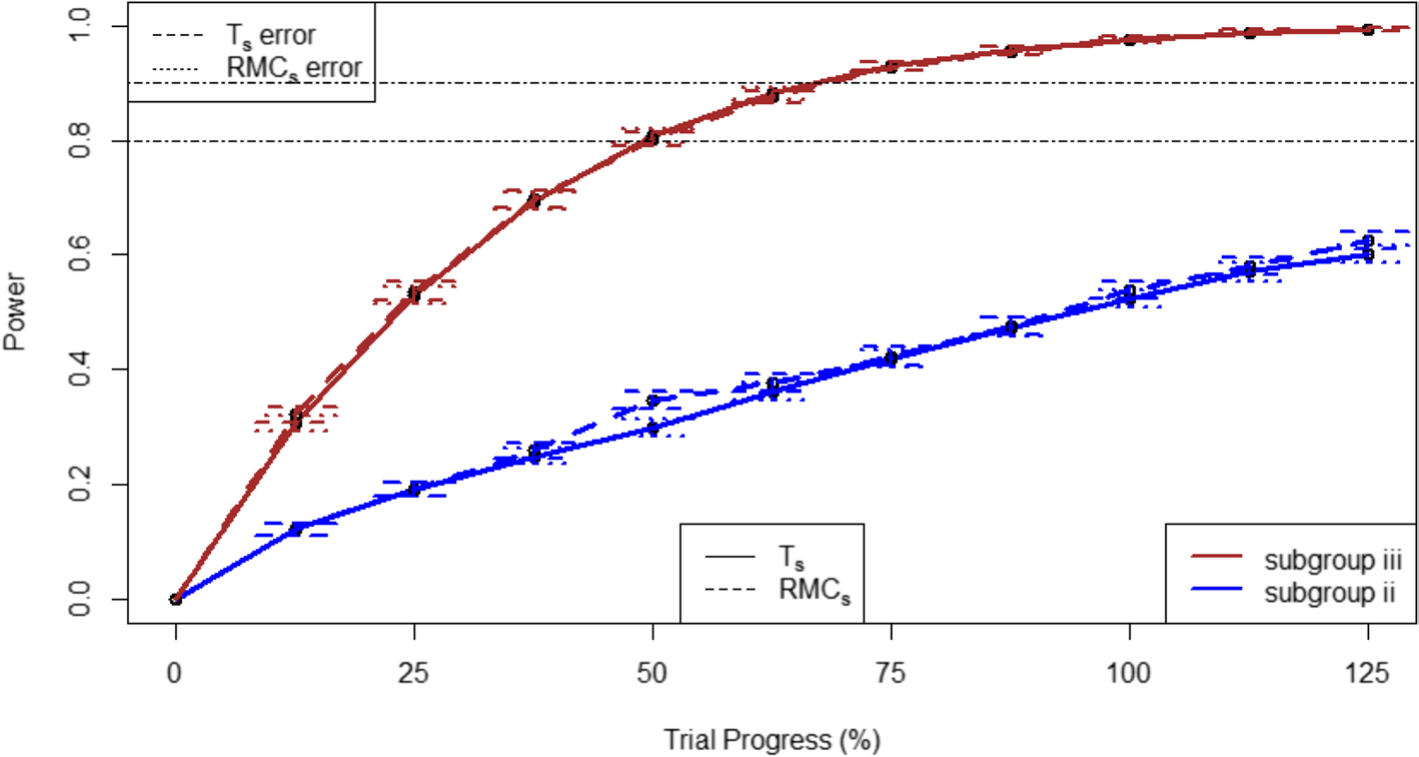
Expected power for subgroups (ii) and (iii) at each trial stage using subgroup-specific adaptive randomisation. Error bars represent represent +/− 1 standard error, with horizontal lines at 80 and 90% power

For subgroup specific RAR procedures, the difference in power is minimal between randomisation approaches; both RMC_s_ and T_s_ produce similar power at all stages. However, there is notable differences between the two subgroups. Subgroup (iii), for whom the treatment effect is largest, has the highest power and reaches 90% power during the study. In contrast, subgroup (ii) doesn’t reach 80% power by the end of the study. This is demonstrated in Fig. 7.

**Fig. 8:**
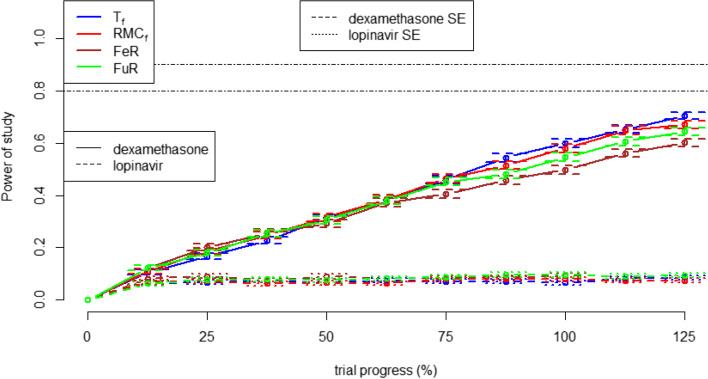
statistical power in determining a significant benefit effect for dexamethasone and lopinavir with different randomisation algorithms. Error bars represent +/− 1 standard error of power, with horizontal lines at 80 and 90% power

In the multi-arm trial simulation, a completely different trend is observed. The RAR allocation methods lead to more power in the dexamethasone and lopinavir arms compared to FuR. FeR, compared to RAR allocation, leads to the more power in the lopinavir arms but less power in the dexamethasone arms. T_f_ allocation leads to slightly more power in both treatment arms compared RMC_f_. These results are demonstrated in Fig. 8.

### Bias and mean squared error in treatment effect estimation

**Fig. 9:**
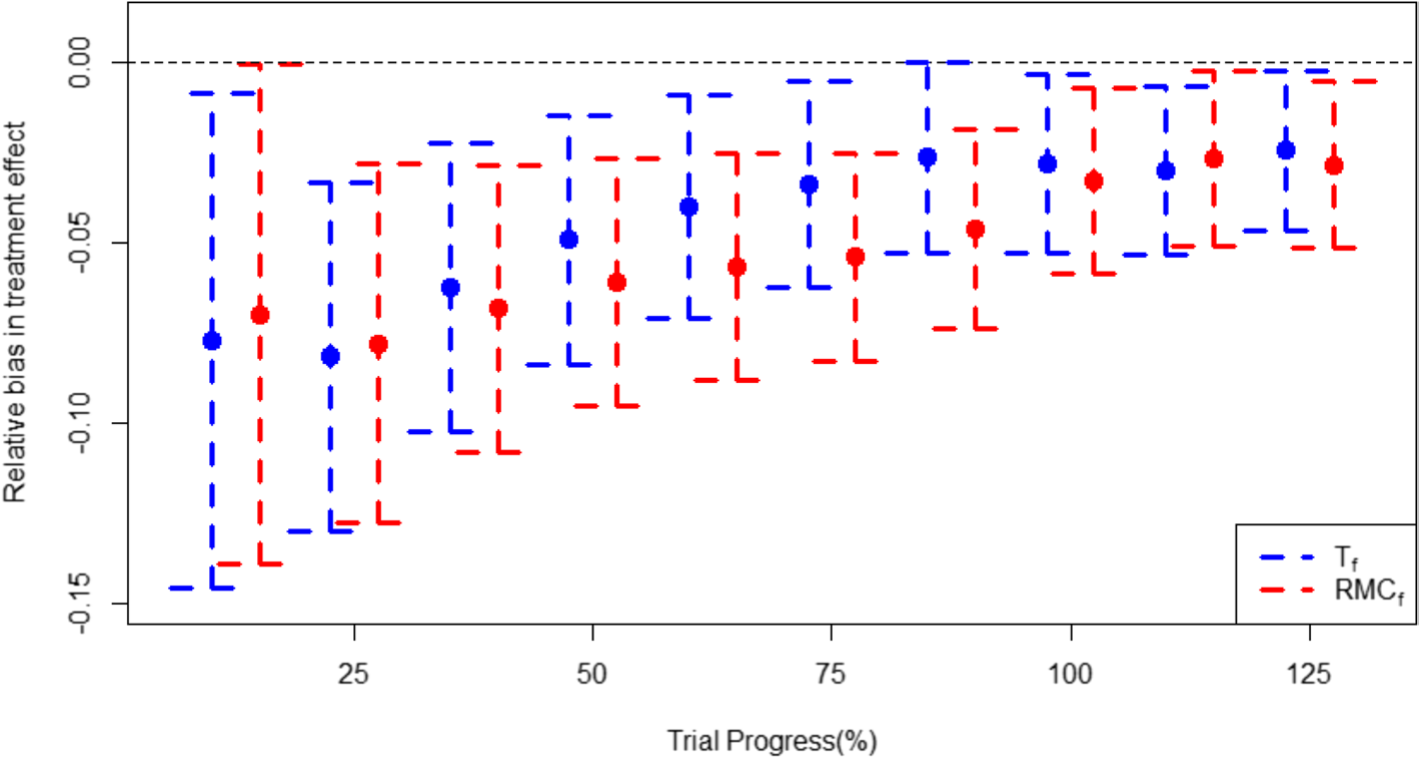
Bias in calculating treatment effect across the trial using the cohort RAR approaches. Error bars represent +/− 1 standard error

**Fig. 10:**
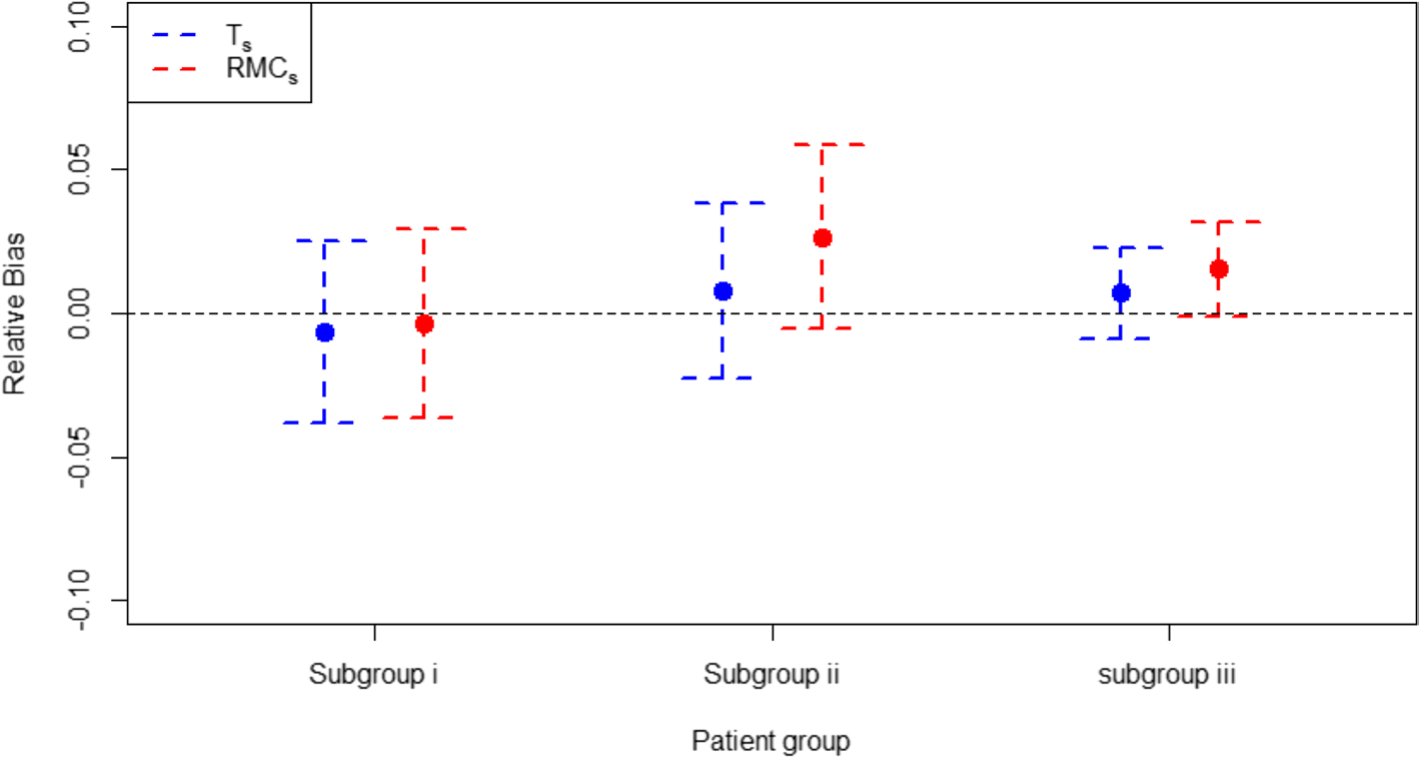
Bias of treatment effect estimates in the full cohort and each subgroup using the four RAR schemes (T_f_, RMC_f_, T_s_, RMC_s_) at the sample size observed in RECOVERY (6425). Error bars represent +/− 1 standard error

It is known that RAR procedures have the potential to induce small sample bias in corresponding treatment effect estimates, because they induce a non-zero correlation between the effect estimate and its sample size [23]. In the two-arm trial setting, the bias associated with using cohort RAR procedures is shown for all stages of the trial in Fig. 9 and in sub-group specific and cohort RAR at the point where the trial reaches the sample size observed in RECOVERY in Fig. 10. Both RMC_f_ is associated with higher bias than T_f_. Sub-group specific RAR results show bias is lowest in subgroup (iii) for both algorithms, with subgroup (i) having the highest bias for the T_s_ algorithm and subgroup (ii) having the highest bias for the RMC_s_ algorithm.

In terms of mean squared error (MSE), FeR leads to the lowest error in the full cohort setting. RMC_f_ leads to higher error than T_f_, with FuR leading to a similar MSE to RMC_f_. This is demonstrated in Table 4. In the subgroup-level randomisation trials, the adaptive randomisation algorithms have a very similar level of MSE in each sub-group, with RMC_s_ leading to higher MSE in subgroup (i) and (iii) and T_s_ leading to slightly higher MSE in subgroup (ii). The MSE in the subgroups negatively correlates with their sample size, with subgroup (ii) having the lowestMSE, followed by subgroup (i) and subgroup (iii). This is demonstrated in Table 2.

**Fig. 11:**
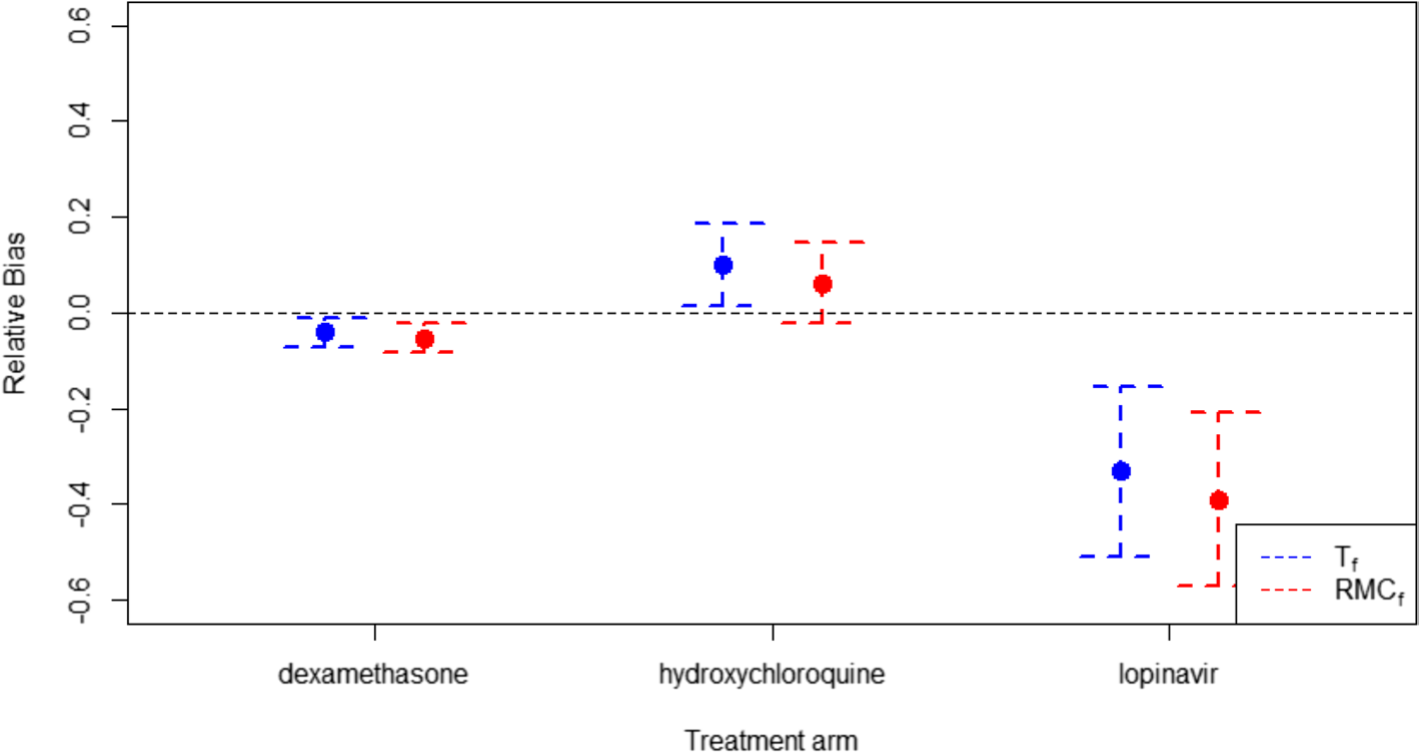
the bias in estimating treatment effect for each of the three experimental treatments in the four-arm trial setting

In four-arm trial setting, treatment effect bias is highest for lopinavir and lowest for dexamethasone. There is very little difference in bias between the T_f_ and RMC_f_ randomisation algorithms, except in the hydroxychloroquine group where T_f_ leads to a small increase in bias. This is shown in Fig. 11. In terms of mean squared error, FuR leads to the highest MSE in all three experimental treatments and FeR leads to the lowest. T_f_ leads to a higher MSE for lopinavir, but otherwise there’s little difference between them. This is demonstrated in Table 3.

### Family wise error rate

**Fig. 12:**
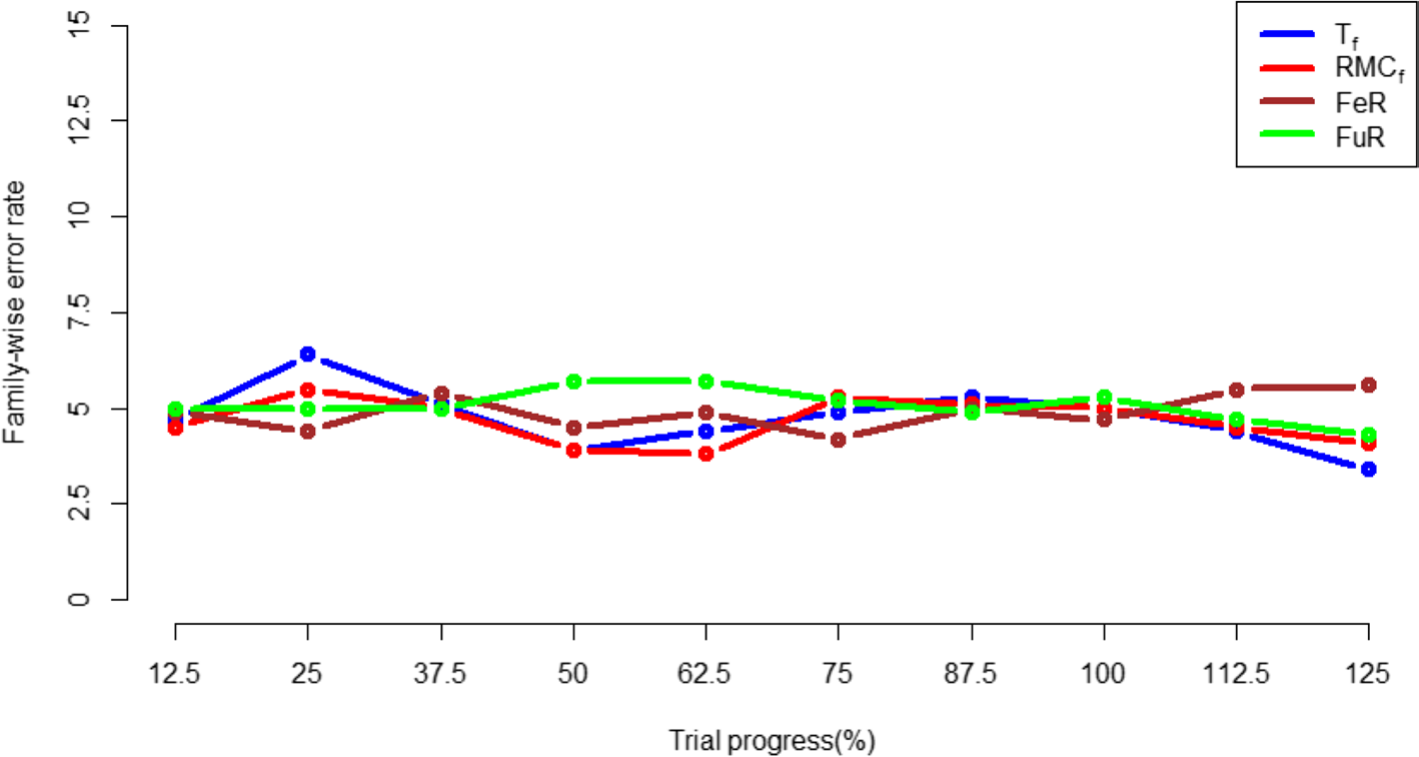
type 1 error rate in the FR and whole-trial RAR approaches for the 2-arm simulation

**Fig. 13:**
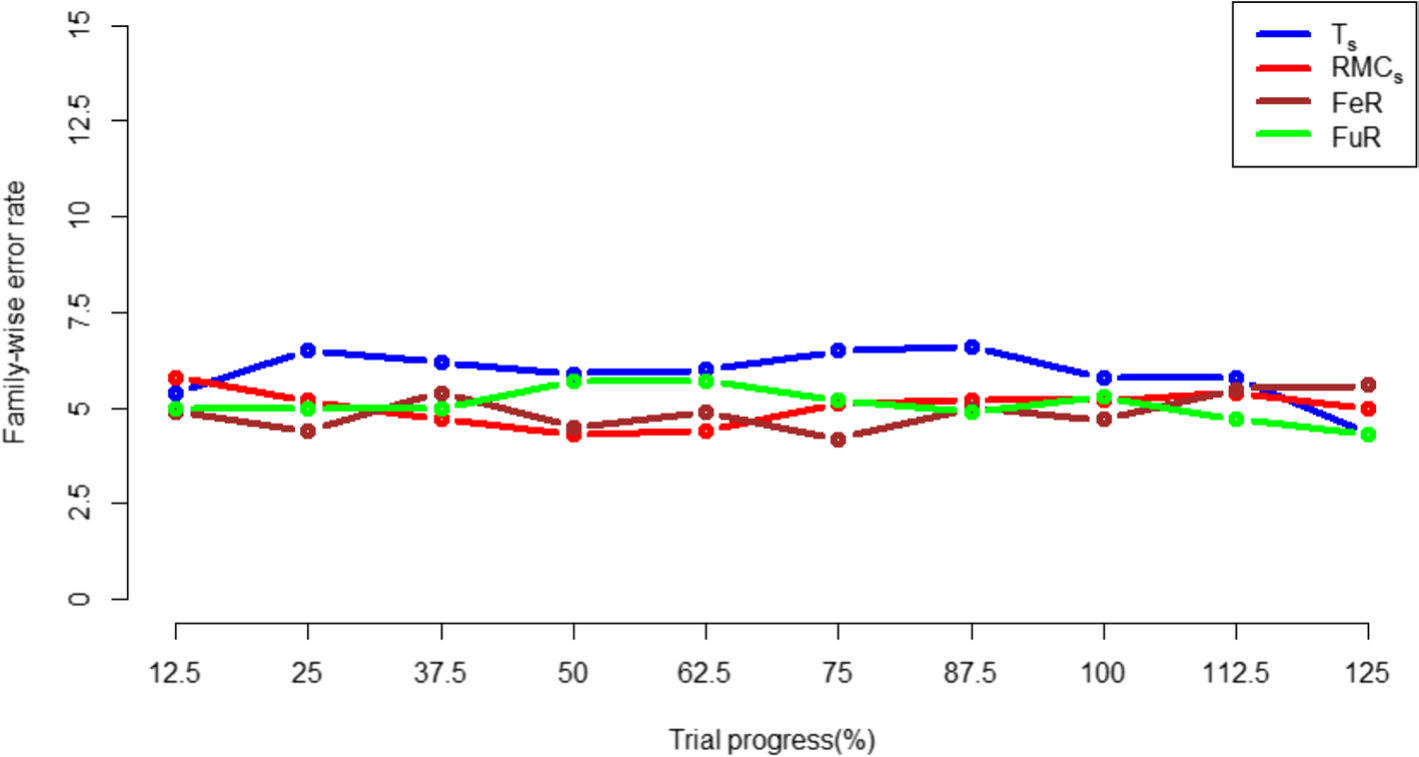
type 1 error rate in the FR znd subgroup-specific RAR approaches in the 2-arm simulation

**Fig. 14:**
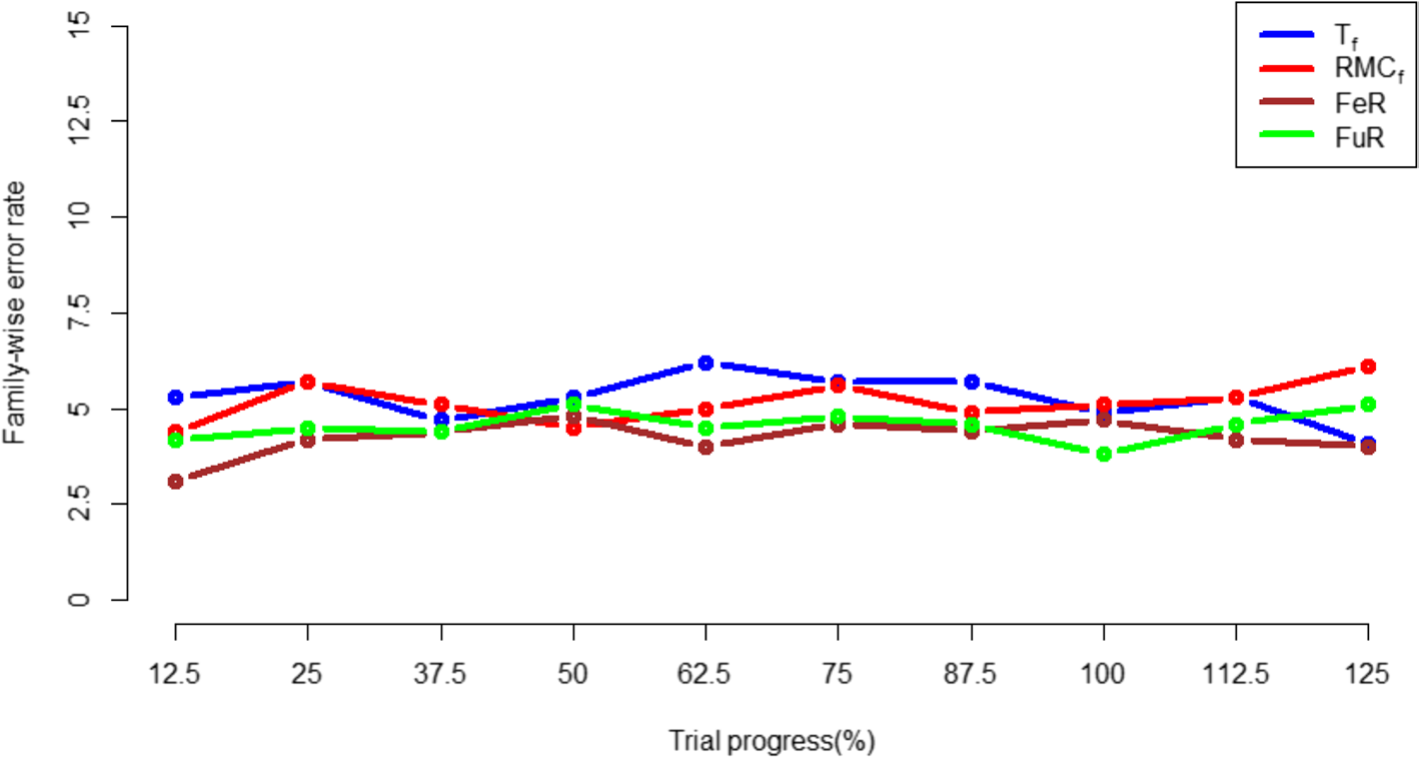
family-wise error rate for all randomisation approaches in the 4-arm simulation

When applying both the whole-trial and subgroup-level randomisation algorithms in the two-arm simulation, the type 1 error rate exhibits small fluctuations around 5%, which is the same as the *p*-value set, as shown in Fig. 12 and Fig. 13 The same occurs when inspecting the Bonferroni corrected family-wise error rates in the 4-arm simulation, as shown in Fig. 14. This demonstrates type-1 errors were not inflated by RAR approaches.

## Discussion

### Understanding the results

It is important to consider why the different approaches led to different mortality rates. Full cohort RAR approaches led to more patients receiving dexamethasone, which was shown to be superior to standard care in most patients, and therefore led to fewer deaths overall in the trial simulation. Subgroup RAR approaches improved mortality even further because they allowed more patients in subgroup (i) to receive standard care and allowed dexamethasone allocation in subgroup (iii) to be ramped up even faster. In the multi-arm simulation, RAR allocation does not reduce mortality by as much. This is because the proportion of patients able to receive the optimal treatment (dexamethasone) is greatly reduced. Forty percent of the allocation is protected to the standard care arm, and the lopinavir and hydroxychloroquine arms will always receive a minimum of 5% of the allocation each. This means the dexamethasone arm can only receive a maximum of 50% of the patients allocated per day, as opposed to 90% in the two arm simulation.

Study power is important because it indicates how many patients are likely to be required to reach statistically significant results. Moreover, in the context of the pandemic, it would mean publishing positive results earlier which would lead to earlier use of the treatment in a real-world context. In the two-arm simulation, FeR achieved the most study power. This is to be expected as Neyman’s allocation formula indicated that the optimal allocation for study efficiency is almost 1:1 [22]. More generally, the power of all FR and cohort RAR strategies (in the two-arm setting) was seen to be inversely proportional to how much their average allocation ratio skewed away from the optimal allocation. Although both FR approaches have more power than the cohort RAR approaches, it is important to mention that FuR, the approach used in the RECOVERY trial, is only marginally more powerful despite leading to the most in-trial deaths. In the four-arm trial simulation, the adaptive randomisation approaches lead to increased power in the dexamethasone group and reduced power for the lopinavir groups. This is consistent with Neyman’s allocation rule, as increasing an experimental arm’s sample size brings it closer to the 1:1 ratio that maximises statistical power.

Even though our results for treatment effect bias may not show a consistent pattern, this is expected with the treatment effect metric, as the magnitude of bias induced in each treatment arm doesn’t follow a predictable pattern [23].

### Wider implications

Our results for the 2-arm simulation illustrate the trade-off in reducing patient deaths within the trial using RAR and getting statistically significant results earlier using FR. However, in the more representative four-arm simulation, not only is the overall mortality rate reduced, but the statistical power in determining the benefit of dexamethasone is increased. This trade-off here is the decreased probability of allocation for the less effective lopinavir group, which would mean it would take longer to halt allocation to this arm as a result of lack of evidence of benefit.. Notwithstanding, given the main role of RECOVERY was to find effective COVID-19 treatments as quickly as possible, this seems like a trade-off that may have been worthwhile to explore. Discovering dexamethasone’s efficacy in managing COVID-19 and the subsequently using of the drug to treat patients has already been estimated to have saved a million lives globally [24]. Furthermore, RAR designs have been shown to improve trial recruitment, precisely because patients understand they have a higher chance of receiving a well-performing treatment [25].. This could have arguably increased the sample size available and thus increase power even further. Article 8 of the World Medical Association Declaration of Helsinki states that the goal of acquiring knowledge must not come before trial participants’ interests [26], but a case can be made that FR procedures do just that. In addition, our simulation shows that we can acquire some knowledge faster if we prioritise which knowledge is more imperative to save lives.

In this simulation, the differences between the tuning algorithm and the REMAP-CAP algorithm are very subtle. As would be expected from their formulas, the tuning algorithm tends to be more exploitative of the superior treatment arms in the later stages of the trial, whereas large recruitment discrepancies between arms in the REMAP-CAP algorithm is self-limiting. In the interests of discovering dexamethasone faster and saving as many lives as possible, the tuning algorithm appears to have the edge. However, it would be less feasible to use in a trial such as RECOVERY as the tuning of it depends on a set timeframe for which to complete the trial. In contrast, the REMAP-CAP algorithm only depends on sample sizes within subgroups and therefore doesn’t have this deficiency.

There is no doubt that the implementation of RAR in a trial is more challenging than FR. This is especially true for subgroup specific RAR. While the subgroups used were declared before the start of the trial, it would be impossible to know that the treatment effect would be different across the three groups, or whether other pre-specified subgroups should have been split for RAR instead. Using RAR at the subgroup level means splitting the sample, leading to smaller sample sizes in each group. This led to a low statistical power for subgroup (i) but a higher power for subgroup (iii) due to the large treatment effect. This meant that the subgroup where the treatment effect is greatest would benefit most from RAR.

### Limitations

One important part of the model that remains unaccounted for is patient drift. Patient drift occurs when the trial cohort’s characteristics, and therefore their likelihood of responding to a treatment, changes throughout the course of a trial [27]. When using FR, the effect of patient drift will be minimised as any changes will be independent of treatment arm allocation (e.g. if patients present later in the trial had fewer comorbidities, both the standard care and dexamethasone groups would have exhibited lower mortality rate). This is not the case when using RAR because arms that performs better will receive a larger proportion of the patients as the trial progresses. Consequently, when calculating response rates, it must be noted that the characteristics of patients could potentially be unbalanced across arms. Throughout the pandemic, data shows the type of people susceptible to catching COVID-19 has changed dramatically. This occurred in terms of age-groups, ethnicities, socio-economic class, and geography [28]. Likewise, the virus itself is likely to have changed, as mutations occur, and as different claves and variants become more common [29]. Resources in treating the pandemic may also change, affecting how likely patients are to survive. For example, the typical care that COVID-19 patients get varies as clinical knowledge in treating the disease improves. Additionally, if more ventilators are procured or there are fewer COVID-19 patients in a hospital, a larger proportion of patients may be placed on ventilators. This has the dual effect of making the full cohort more likely to survive as more patients can receive adequate respiratory support, and of diluting the ventilator sub-group with healthier patients as ventilators do not have to be reserved for only the most critical patients. Therefore, had RAR been applied, it is likely the death rate would be skewed by the confounding effect of difference in patient, illness, and management characteristics.

Additionally, like all adaptive trial designs, implementing RAR creates certain operational challenges. In RECOVERY, these can be split into challenges which might have prolonged the design of the trial, and challenges which would affect the way it runs. The RECOVERY trial was famously set up and began recruiting patients very quickly, taking just 9 days from the first draft of the protocol to enrolment of the first participant [30]. In contrast, implementing RAR might have added extra hurdles in terms of planning the trial and might have therefore delayed patients being recruited. For example, the varying randomisation ratios require a central system for randomisation and mean that it is harder to predict the drug supply required for each arm. Crucially, this could have counteracted RAR’s ability to attain study power earlier in the trial, meaning it may not have saved lives. Nonetheless, in terms of running the trial, many of the requirements that would allow it to perform response-adaptive randomisation have already been met. RECOVERY had a data monitoring committee which would be needed for RAR. Its heavy use of re-purposed drugs reduces the need for safety monitoring, and its primary endpoint being measured at 28 days allows RAR to be implemented from an early point in the trial [31], as shown in our simulations. Arguably the biggest problem the addition of RAR might pose is the requirement for timely data collection. Given the NHS was lacking resources at certain points in the pandemic [32], it might have been challenging for clinical staff to find the time to log trial patients and their outcomes promptly. It would also have been difficult to arrange additional staff on site to help run the trial, given strict infection control protocols in hospitals.

Also notable is the simplicity of the simulation. RECOVERY has arms being dropped and added dynamically. In contrast, we simulated it as fixed-arm trials with no treatments being dropped or added. Furthermore, for simplicity in the simulation, patient outcomes are generated in uniform batches. This contrasts with what happened in RECOVERY as, the number of hospitalised patients available to recruit varied significantly throughout the trial period, and this would have affected how RAR worked. There was a sharp decrease in hospitalised patients towards the end of the recruitment period [33]. This would have likely meant that there would be more information attained at the start of the trial, and therefore the proportion of patients randomised to dexamethasone would have increased faster than it did in the simulation. Although our simulation study was simplistic, we believe the results paint a broadly accurate picture of how the operating characteristics of RECOVERY would differ using FR and RAR procedures.

### Future directions

To understand RAR within an adaptive platform trial context, further simulation studies could be undertaken to implement more dynamic features of a simulation study. For example, simulations could emulate arms being dropped once there is sufficient confidence to classify whether they are better, the same or worse than standard care. Additionally, simulations could be made more complex to adjust for varying recruitment rates and evaluate the influence of patient drift. Finally, surveys of patients previously hospitalised with covid-19 could be conducted to ascertain attitudes to RAR.

## Conclusion

Using RAR within RECOVERY could have resulted in more patients being given the optimal treatment, and therefore fewer deaths in the trial. These benefits of RAR were even more pronounced when used within pre-specified subgroups. In addition, fewer patients would have been required to attain the same study power under RAR, leading to a shorter trial period (assuming the same recruitment rate). Bias in treatment effect estimation arises in RAR trials, but only to a negligible extent. The use of RAR deserves to be considered for use in future platform trials. The consideration of the needs of patients within and beyond the trial should be acknowledged by trialists more clearly, and patient groups themselves consulted before deciding what balance to strike.

## Supplementary Information

**Additional file 1.**

## Data Availability

The datasets generated and/or analysed during the current study are available in the github repository, https://github.com/ts482/RECOVERY_RAR

## Abbreviations

RAR: Response Adaptive Randomisation
FR: Fixed Randomisation
COVID-19: Coronavirus disease 2019
RECOVERY: Randomised Evaluation of COVID-19 Therapy
REMAP-CAP: Randomised, Embedded, Multi-factorial Adaptive Platform Trial for Community Acquired Pneumonia
FeR: Fixed equal Randomsation
FuR: Fixed unequal Randomisation
T_f_: Tuning protocol on the full patient cohort
RMC_f_: REMAP-CAP randomisation protocol on the full patient cohort
T_s_: Tuning protocol on each subgroup individually
RMC_s_: REMAP-CAP randomisation protocol on each subgroup individually
