# Supplementary material for: Should RECOVERY have used response adaptive randomisation? Evidence from a simulation study
Source: BMC Med Res Methodol. 2022 Aug 6;22:216. doi: 10.1186/s12874-022-01691-w (PMC9356442; doi:10.1186/s12874-022-01691-w)
Supplement: Supplementary file 1 — Additional file 1. [file 12874_2022_1691_MOESM1_ESM.docx]

**Technical appendix**

**Technical Appenix A: Neyman rule for optimal allocation**

Neyman allocation is a procedure that maximises study power for two arms given their estimated binary response rates (1). Rosenberger et al. (1) shows that optimal allocation ratio to treatment 1 for maximising power can be given by:$\frac{\sqrt{\hat{P}_{1}}}{\sqrt{\hat{P}_{1}}+ \sqrt{\hat{P}_{0}}}$. In the case of the RECOVERY trial, $\hat{P}_{1}$is the mortality rate on dexamethasone (22.9%) and $\hat{P}_{0}$is the observed mortality rate on standard care (25.7%). Substituting these into the equation indicates that 49% of patients should receive dexamethasone to maximise the power of the study. Substituting the mortality rates on each treatment for the subgroups i, ii and iii (found in Table 1), the optimal allocation ratios to dexamethasone would be 53%, 49% and 46% respectively.

**Technical Appendix B: Calculating the posterior outcome probability**

To calculate the posterior probability that one treatment arm yields superior results to the other, a Bayesian beta-binomial model was assumed for each trial arm. The prior beta distribution can be given as $\pi\left( p | \alpha,\beta\right)= \frac{1}{B(\alpha,\beta)}p^{\alpha-1}{(1-p)}^{\beta-1}$, where $B\left( \alpha, \beta\right)= \int_{0}^{1} p^{\alpha-1}{(1-p)}^{\beta-1}dp$ and represents the Beta function. α and β are set to one. The binary outcome of each patient is mortality within 28 days after randomization and can be regarded as an independent Bernoulli trial. Therefore, a sample of n patients follows a binomial distribution, such that $f\left( y | p \right)= \binom{n}{y} p^{y}{(1-p)}^{n-y}$, where n is the total number of patients and y is the number of successes (or deaths within 28 days of randomization). Combining these distributions creates the posterior probability density function π(p│y), given as $\frac{1}{B\left( y+\alpha, n-y+\beta\right)}p^{y+\alpha-1}{(1-p)}^{n-y+\beta-1}$, which is a beta(y+ α, n - y + β) distribution.

The parameters indexing the posterior beta distribution are therefore $\alpha=y+\alpha$ and $\beta=n-y+\beta$. From this we can create a beta posterior distribution at each point of the interim analysis *i* = 1,…,100 for each trial arm *j* = 0 (dexamethasone), 1 (standard care), and 100 points *k* = 1,…,100. To estimate, for example, the posterior probability that the posterior mean for arm 0 is greater than the posterior mean of arm 1, we randomly generate 100 draws from each distribution and count the proportion of times that the arm 0 draw is the largest.

**Technical Appendix C: Bowden and Trippa rule for bias following a RAR procedure**

In general, FR schemes lead to unbiased estimates of the treatment effect. Conversely, RAR procedures induces bias into the treatment effect estimate, because the sample size of the trial arm becomes positively correlated with the estimate itself (2). Specifically, the bias of a proportion parameter is given by:

$$\frac{Cov\left[ n_{j}{, \hat{P}}_{j} \right]}{E\left[ n_{j} \right]}=P_{j}-E\left[ \hat{P}_{J} \right]= -Bias(\hat{P}_{j})$$

Where n_j_ is the sample size of treatment arm j, P_j_ is the true mortality rate in treatment arm j, and ${, \hat{P}}_{j}$ is the estimated mortality rate in treatment arm j based on trial data.

References:

1. Rosenberger WF, Stallard N, Ivanova A, Harper CN, Ricks ML. Optimal Adaptive Designs for Binary Response Trials. Biometrics. 2001;57(3):909–13.

2. Bowden J, Trippa L. Unbiased estimation for response adaptive clinical trials. Stat Methods Med Res. 2017 Oct;26(5):2376–88.
